# Roles of Sestrin2 and Ribosomal Protein S6 in Transient Global Ischemia-Induced Hippocampal Neuronal Injury

**DOI:** 10.3390/ijms161125963

**Published:** 2015-11-04

**Authors:** Yao-Chung Chuang, Jenq-Lin Yang, Ding-I Yang, Tsu-Kung Lin, Chia-Wei Liou, Shang-Der Chen

**Affiliations:** 1 Department of Neurology, Kaohsiung Chang Gung Memorial Hospital, College of Medicine, Chang Gung University, Kaohsiung 833, Taiwan; ycchuang@adm.cgmh.org.tw (Y.-C.C.); tklin@adm.cgmh.org.tw (T.-K.L.); cwliou@ms22.hinet.net (C.-W.L.); 2 Center for Translational Research in Biomedical Sciences, Kaohsiung Chang Gung Memorial Hospital, Kaohsiung 833, Taiwan; jyang@adm.cgmh.org.tw; 3 Department of Neurology, Faculty of Medicine, College of Medicine, Kaohsiung Medical University, Kaohsiung 833, Taiwan; 4 Institute of Brain Science, National Yang-Ming University, Taipei 112, Taiwan; diyang@ym.edu.tw

**Keywords:** transient global ischemia, hippocampus CA1 neurons, oxidative stress, ribosomal protein S6, sestrin2

## Abstract

Recent studies suggested that sestrin2 is a crucial modulator for the production of reactive oxygen species (ROS). In addition, sestrin2 may also regulate ribosomal protein S6 (RpS6), a molecule important for *protein synthesis,* through the effect of mammalian target of rapamycin (mTOR) complex that is pivotal for longevity. However, the roles of sestrin2 in cerebral ischemia, in which oxidative stress is one of the major pathogenic mechanisms, are still less understood. In this study, we hypothesized that sestrin2 may protect hippocampal CA1 neurons against transient global ischemia (TGI)-induced apoptosis by regulating RpS6 phosphorylation in rats. We found that sestrin2 expression was progressively increased in the hippocampal CA1 subfield 1–48 h after TGI, reaching the maximal level at 24 h, and declined thereafter. Further, an increased extent of RpS6 phosphorylation, but not total RpS6 protein level, was observed in the hippocampal CA1 subfield after TGI. The *sestrin2* siRNA, which substantially blocked the expression of TGI-induced sestrin2, also abolished RpS6 phosphorylation. TGI with reperfusion may induce oxidative stress with the resultant formation of 8-hydroxy-deoxyguanosine (8-OHdG). We found that *sestrin2* siRNA further augmented the formation of 8-OHdG induced by TGI with reperfusion for 4 h. Consistently, *sestrin2* siRNA also enhanced apoptosis induced by TGI with reperfusion for 48 h based on the analysis of DNA fragmentation by agarose gel electrophoresis, DNA fragmentation sandwich ELISA, and the terminal deoxynucleotidyl transferase-mediated dUTP-biotin nick end labeling (TUNEL) assay. Together these findings indicated that TGI-induced sestrin2 expression contributed to RpS6 phosphorylation and neuroprotection against ischemic injury in the hippocampal CA1 subfield.

## 1. Introduction

Sestrins belong to a family of highly conserved proteins initially discovered as p53-inducible proteins and are believed to protect cells against various insults [1]. Mammalian cells express three isoforms of sestrins, namely sestrin1, sestrin2, and sestrin3 [2,3,4]. Several mechanisms have been proposed to be responsible for the protective roles of sestrins. One is that sestrins can function as an antioxidant to scavenge excessive reactive oxygen species (ROS) [3]. The other mechanism is that, under a stressful condition, p53-induced sestrins may activate AMP-dependent protein kinase (AMPK) accompanied by suppression of mammalian target of rapamycin (mTOR), leading ultimately to autophagy induction [5]. Sestrin2, also known as hypoxia-inducible gene95 (Hi95), is vital for metabolic stability under prolonged hypoxia [3]. Sestrin2 is an acute response protein and plays a crucial role under various conditions such as excessive oxidative stress, DNA damage, hypoxia, mitochondrial dysfunction, and amyloid beta-induced neurotoxicity [6,7,8,9,10]. However, the study of sestrin2 in cerebral ischemia is limited. In an experimental model of acute stroke, sestrin2 was highly induced in the cortical area based on the histological study, suggesting a potentially important role of sestrin2 in the ischemic brain [1]. These results may denote the pivotal role of sestrin2 in tissues under ischemic or hypoxic conditions that may be worthy of further exploration.

Ribosomal protein S6 (RpS6) is an evolutionarily conserved protein that spans 236–253 residues in various species including yeast, plants, invertebrates, and vertebrates [11]. The essential role of ribosomes is protein translation and it is, therefore, thought to be involved in regulation of protein synthesis. RpS6 is critically implicated in liver regeneration, thymus gland development, and T-cell maturation in peripheral lymphoid organs [12]. RpS6 may be subjected to phosphorylation under various physiological, pathological, and pharmacological stimuli [11]. The physiological roles of RpS6 phosphorylation include global protein synthesis, translational control of mRNAs, cell proliferation, and glucose homeostasis [11]. In a recent study [13], it was demonstrated that ischemic preconditioning results in the phosphorylation of RpS6 in mouse hearts, which exerts protective effects against oxidative stress that can be disrupted by the *RpS6* siRNA.

Recently, emerging evidence suggested that, under stressful conditions, the sestrin2 signaling pathway involves p53, AMPK, and mTOR [14,15] as well as mTOR-regulated RpS6 expression [16]. It is thus reasonable to speculate that, under cerebral ischemic insult, sestrin2 and RpS6 may exert protective effects to counteract the detrimental effect of ischemia and decrease neuronal injury. However, the potential link between sestrin2 and RpS6 in cerebral ischemia has never been reported before. In the present study, we therefore investigated the hypothesis that the sestrin2 signaling pathway plays a protective effect in the hippocampal CA1 subfield in transient global ischemia (TGI)/reperfusion through the regulation of RpS6 phosphorylation.

## 2. Results and Discussion

### 2.1. Temporal Changes of Sestrin2 and RpS6 Expressions in the Hippocampal CA1 Subfield after TGI

**Figure 1:**
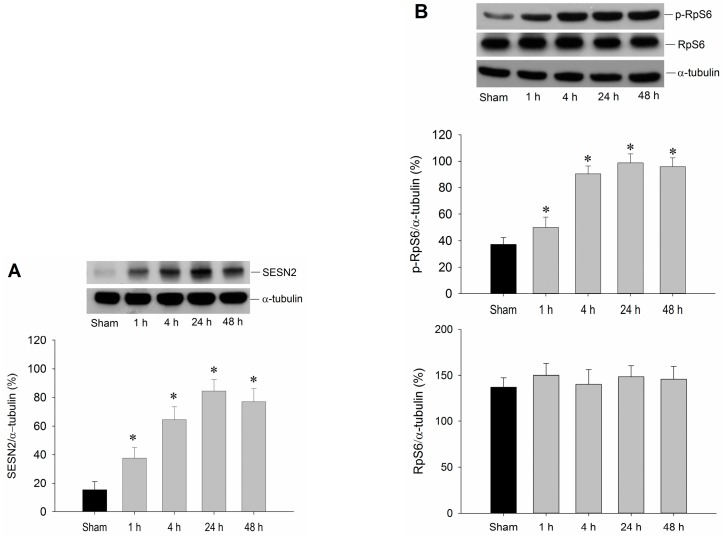
Transient induction of sestrin2 and RpS6 by TGI/reperfusion. The rats were under a 10 min of TGI followed by reperfusion for indicated times. Sham-operated animals served as negative controls. Hippocampal CA1 samples were then collected for Western blotting to detect expression levels of sestrin2 (**A**) as well as RpS6 and p-RpS6 (**B**). The blots were also re-probed with an anti*-*α*-*tubulin antibody as a loading control *i*n each lane. Values are mean ± SEM from representative blots and quantitative analyses from five to seven animals in each experimental group are shown. * *p* < 0.05 *vs.* sham control group in the Scheffe multiple-range test. SENS2: sestrin2.

We first examined whether sestrin2 was induced by TGI in the hippocampal CA1 subfield. Protein immunoblot showed an evident increase of sestrin2 expression in the hippocampal CA1 subfield 1–48 h after TGI, reaching the maximal level at 24 h (Figure 1A). It was reported that the sestrin2 signaling pathway involves mTOR [14,15], and mTOR may regulate RpS6 expression [16]. Emerging evidence also revealed that RpS6 plays a key role in cardiac protection under ischemia [13]. It is therefore intriguing to know whether the expression of RpS6 is affected by TGI in the hippocampal CA1 subfield. The results shown in Figure 1B indicated that TGI with reperfusion up to 48 h failed to affect the expression of total RpS6; however, a progressive augmentation of RpS6 phosphorylation (p-RpS6) was detected in rat hippocampal CA1 regions in 1–48 h after TGI. Thus, TGI/reperfusion selectively induces p-RpS6, but not the total RpS6 expression, in the CA1 subfield of the hippocampus in rats.

### 2.2. Sestrin2 siRNA Silences Sestrin2 Expression and Diminishes p-RpS6 Expression in the Hippocampal CA1 Subfield after TGI

**Figure 2:**
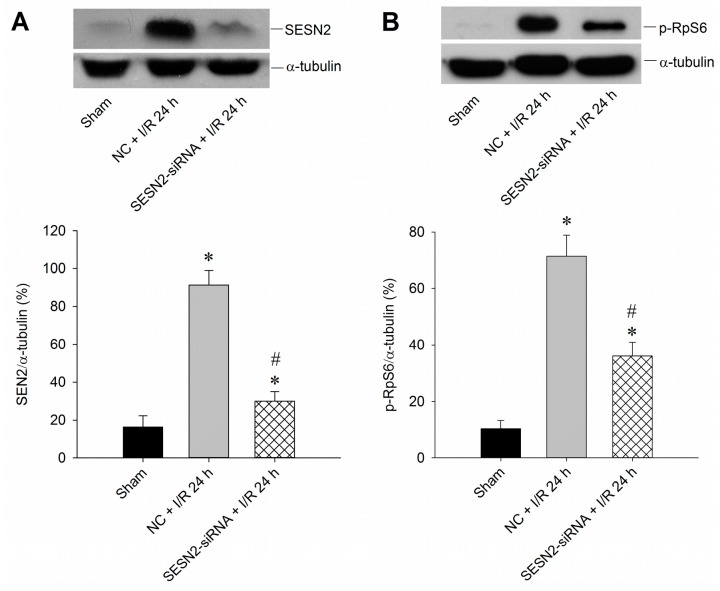
*Sestrin2* siRNA decreases expression of both sestrin2 and p-RpS6 in the hippocampal CA1 subfield after TGI/reperfusion. (**A**) Rats were microinjected into bilateral CA1 subfields with control siRNA or *sestrin2* siRNA 24 h before TGI. Total proteins of the hippocampal CA1 subfield were collected from the sham-operated controls without siRNA microinjection (Sham), and the rats injected with negative control siRNA (NC + I/R 24 h) or the *sestrin2* siRNA (SESN2 + I/R 24 h), followed by 10 min of TGI and 24 h of reperfusion before detection of sestrin2 expression; (**B**) The experimental condition was the same as in (A) except that the detection of p-RpS6 was conducted. In both (**A**) and (**B**), the same blots were also re-probed with an anti-α-tubulin antibody to serve as a loading control in each lane. Values are mean ± SEM from four to six animals per experimental group. * *p* < 0.05 *vs.* sham-control group; ^#^ *p* < 0.05 *vs.* negative control siRNA + I/R in the Scheffe multiple-range test. I/R: ischemia/reperfusion. NC: negative control siRNA. SENS2: sestrin2.

To further clarify the pivotal roles of sestrin2 in this ischemic paradigm of the brain, a molecular approach by microinjecting *sestrin2* siRNA bilaterally into the hippocampal CA1 subfields was adopted to elucidate the underlying mechanisms. Results showed that *sestrin2* siRNA successfully down-regulated sestrin2 expression in the hippocampal CA1 subfield after TGI (Figure 2A). As sestrin2 signaling may regulate p-RpS6 expression [14,15,16], we therefore tested whether the suppression of sestrin2 may affect p-RpS6 expression in ischemia/reperfusion. Results indicated that the *sestrin2* siRNA reduced the levels of p-RpS6 that were induced by 10 min of TGI with 24 h of reperfusion (Figure 2B), suggesting that TGI-induced sestrin2 contributed to the expression of p-RpS6.

### 2.3. Injection of Sestrin2-siRNA Augments Oxidative Stress and Increases Neuronal Injury in the Hippocampal CA1 Subfield after TGI

**Figure 3:**
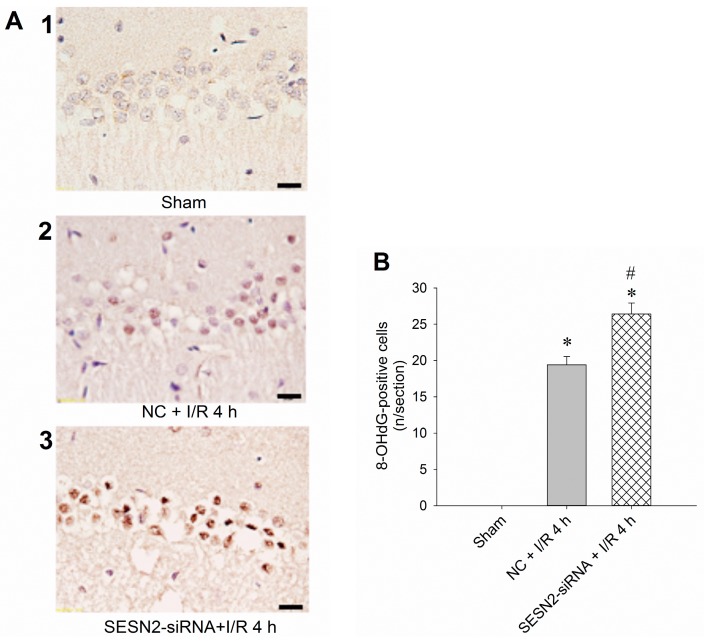
*Sestrin2* siRNA augments oxidative stress in the hippocampal CA1 subfield after TGI. (**A**) Rats were microinjected with negative control siRNA (NC + I/R 4 h) or the *sestrin2* siRNA (*sestrin2* siRNA + I/R 4 h) into the CA1 subfield 24 h before 10 min of TGI with reperfusion for 4 h. The sham-operated controls without siRNA microinjection served as controls (Sham). The hippocampal CA1 subfield was subjected to immunohistochemistry for quantitative assessments of 8-OHdG as an index for oxidative DNA damage; (**B**) Values are shown as mean ± SEM from four to six animals in each experimental group. * *p* < 0.05 *vs.* sham control group and ^#^ *p* < 0.05 *vs.* negative control siRNA + I/R in the Scheffe multiple-range test. Scale bar: 20 μm. I/R: ischemia/reperfusion. NC: negative control siRNA. SENS2: sestrin2.

**Figure 4:**
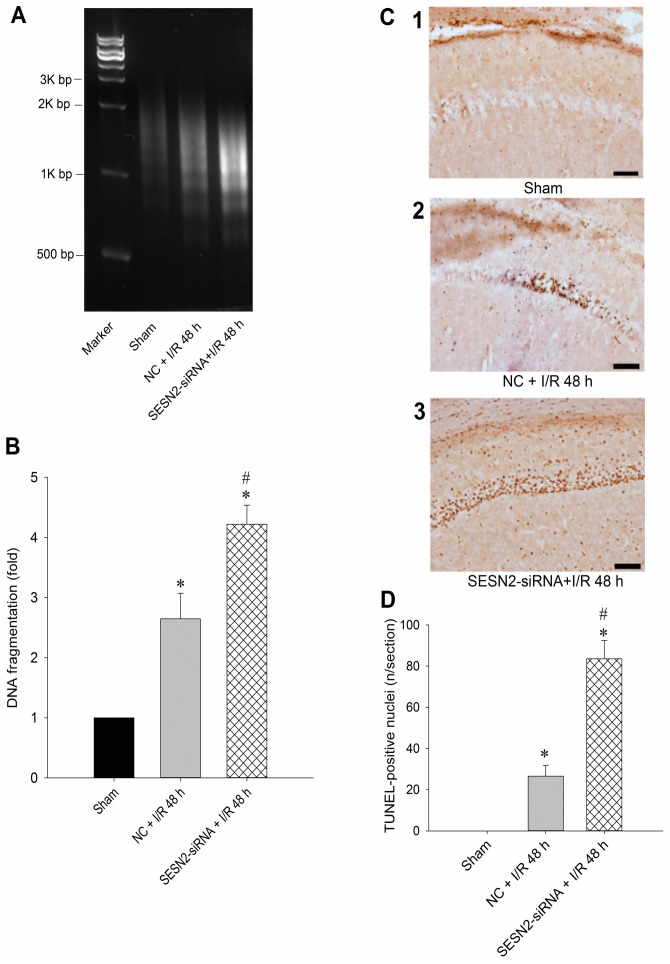
*Sestrin2* siRNA augments apoptosis-related neuronal damage in the hippocampal CA1 subfield after TGI. The experimental protocol was the same as described in Figure 3 except that the reperfusion time was 48 h. (**A**) The genomic DNA was subjected to PCR assay to reveal apoptosis-related DNA fragmentation by agarose gel electrophoresis; (**B**) The tissue homogenates of the hippocampal CA1 subfields were subjected to DNA fragmentation sandwich ELISA to assess histone-associated DNA fragments in the cytoplasm as a quantitative measure for the DNA fragmentation; (**C**,**D**) Hippocampal slices were subjected to TUNEL staining to resolve the extents of apoptosis. Values in (**B**,**D**) are mean ± SEM from five to seven animals in each experimental group. * *p* < 0.05 *vs.* sham control group and ^#^ *p* < 0.05 *vs.* negative control siRNA + I/R in the Scheffe multiple-range test. Scale bar in (**C**): 100 μm. I/R: ischemia/reperfusion. NC: negative control siRNA. SENS2: sestrin2.

To further clarify the pivotal role of sestrin2 in this ischemic paradigm of the brain, we investigated the effects of *sestrin2* siRNA over TGI-dependent oxidative stress and apoptosis. We found that pretreatment with *sestrin2* siRNA significantly augmented the extent of DNA oxidation, as evidenced by the increased numbers of 8-OHdG-positive cells in the hippocampal CA1 subfield 4 h after onset of reperfusion (Figure 3A); quantitative data confirmed this finding (Figure 3B). These findings are compatible with our previous results of quantifying the levels of 8-OHdG using a DNA oxidation kit [17]. Furthermore, the suppression of sestrin2 by siRNA further enhanced the extents of hippocampal neuronal apoptosis based on various experimental approaches. These included agarose gel electrophoresis to detect the extents of DNA fragmentation (Figure 4A), sandwich DNA fragmentation ELISA to quantitatively determine the amounts of oligonucleosomes in hippocampal tissue homogenates (Figure 4B), and TUNEL staining with quantitative data (Figure 4C,D) to detect apoptosis, all within 48 h after ischemia/reperfusion, as has been reported previously [17,18,19]. Taken together, these findings revealed that inhibition of sestrin2 expression may enhance oxidative damage and apoptosis-related DNA fragmentation in the hippocampal CA1 subfield after TGI.

### 2.4. Discussion

In the present study, we showed that sestrin2 expression was enhanced in the hippocampal CA1 subfield after TGI. An elevated level of p-RpS6, which is important for protein synthesis and may possess protective effects under the ischemic condition, was also detected in the hippocampal CA1 subfield after TGI. Down-regulation of sestrin2 by siRNA resulted in the decreased extent of p-RpS6 in the hippocampal CA1 subfield after TGI. Attenuation of sestrin2 expression by siRNA also increased the extents of DNA oxidation and apoptosis in hippocampal CA1 neuronal cells. Together, these results demonstrate that TGI can induce the expression of sestrin2 in the hippocampal CA1 subfield, which may represent an endogenous protective mechanism in response to cerebral ischemia in part through regulation of p-RpS6.

In the current study, the physiological parameters (*i.e.*, pH, pCO_2_, pO_2_, mean arterial blood pressure, *etc.*) before and after the induction of TGI were not provided. Nevertheless, our protocols for TGI were based on the previously reported model [20]. In that TGI procedure, all the physiological parameters, including pH, pCO_2_, pO_2_, and mean arterial blood pressure, were not different before and after the TGI procedures among different sets of mean arterial blood pressure from 36–40 mmHg (mild hypotension group), 31–35 mmHg (moderate hypotension group), to 26–30 mmHg (severe hypotension group) [20]. Given our previous publications [17,18,19,21], together with the oxidative damages of the hippocampal CA1 subfield shown in the present study, this is a feasible model for exploring the molecular mechanisms underlying TGI-induced hippocampal neuronal injuries.

Selective neuronal loss in the hippocampal CA1 subfield is a histological feature of TGI and reperfusion which occurs days after the initial ischemic insult [22]. Despite many theories and efforts to explain this postponed neuronal death in the CA1 subfield of the hippocampus, the underlying mechanisms remain unclear. Common pathogenic mechanisms, including impaired mitochondrial function, increased oxidative damage, excitotoxicity, and inflammation, are all implicated in various ischemic conditions [23,24]. It was suggested that ischemic neuronal damage is mainly due to an inflammatory pathogenesis. The inflammatory process is engaged in all phases of the ischemic cascades, from the early detrimental events triggered by arterial occlusion to the late restorative processes underlying post-ischemic tissue repair, and both the innate and adaptive immune response may offer an innovative therapeutic strategy [25,26]. Activation of inflammation-related signaling pathways in ischemic stroke includes the Toll-like receptors pathway, the mitogen-activated protein kinases pathway, and the nuclear factor-kappa B pathway [27]. In addition to inflammatory responses that play the main role in ischemic pathogenesis, the crucial participant of oxidative stress is well known in cerebral ischemia [23,24,28]. Drugs and chemical compounds possessing the characters of anti-oxidative stress can exert beneficial effects under ischemic insults [29,30]. Notably, inflammation and oxidative stress are mutually implicated with each other. For example, superoxide anion produced by the NADPH oxidase in activated microglia may interact with nitric oxide (NO) that is generated from inflammatory responses to form the active oxidant peroxynitrite [31]. In the current study, we revealed the crucial roles of sestrin2 in attenuating oxidative damage caused by TGI/reperfusion. However, whether the observed sestrin2 effect may also link to the attenuation of inflammatory responses requires further investigation.

Sestrins are stress-inducible proteins capable of protecting cells against various insults [1]. Among them, sestrin2 appears to be a stress sensor that is protective against stress-induced apoptosis and incorporates the pro-survival function of Akt and the negative regulation of mTOR [14,32]. It has been shown that sestrin2 ablation aggravates obesity-induced mTORC1-S6K pathways, glucose intolerance, insulin resistance, and hepatosteatosis [33]. These results reveal an important homeostatic function of sestrin2 in the control of lipid and glucose metabolism [33]. Emerging evidence also suggests that, under stressful conditions, sestrin2 expression involves the AMPK and mTOR-S6K pathways [15]. While the exact function of sestrin2 is yet to be clarified, and sestrin2 may control intricate cellular functions in response to different stress conditions [1], evidence from various ischemic conditions suggests that sestrin2 may possess a protective role under cerebral ischemia [34,35]. It was reported that renal ischemia-reperfusion injury up-regulated the expression of sestrin2 in the proximal tubules to protect the renal tubules from acute kidney injury [34]. In a recent study, sestrin2 exerted a cardioprotective effect against ischemia/reperfusion injury, serving as an LKB1-AMPK scaffold to commence AMPK activation during ischemic insults [35]. Consistent with these findings, in this study we showed that TGI increases sestrin2 expression in the hippocampal CA1 subfield with a progressive augmentation from 1–48 h that peaked at 24 h after reperfusion (Figure 1A). This finding may denote a potential role of sestrin2 in this ischemic condition. Although mTOR may regulate protein translation, which is important for cellular growth and proliferation, reports about mTOR are conflicting, with either protective or detrimental effect in ischemia/reperfusion [36,37,38]. In a recent ischemic preconditioning study of the heart that involved mTOR expression, p-RpS6 represented a convergent point for cardioprotection [13]. As the sestrin2 signaling pathway involving mTOR [14,15] and mTOR may regulate RpS6 expression [16], it is tempting to speculate the inter-relationship between sestrin2 and RpS6 under TGI. In this study, we showed a significant change of p-RpS6, but not the total RpS6, in the hippocampal CA1 subfield after TGI, which suggests an important role of p-RpS6 in this ischemia paradigm (Figure 1B). These results are in line with the contention that sestrin2 expression and p-RpS6 may have a potentially protective effect in the hippocampal CA1 subfield in TGI/reperfusion.

To show the pivotal role of sestrin2 in this ischemic paradigm, we used *sestrin2* siRNA to silence its expression in the hippocampal CA1 subfield after TGI (Figure 2A). Concomitantly, the extent of p-RpS6 decreased with *sestrin2* siRNA treatment (Figure 2B). RpS6, known to be vital for the regulation of protein synthesis [11], may be important to counteract oxidative stress under an ischemic condition with phosphorylated states. It was known that an excessive oxidative stress underlies neuronal cell damages in the CA1 subfield of the hippocampus after TGI [17,19,21]. The extent of TGI-induced oxidative neuronal damage is linked to the expression of sestrin2, which is based on the findings that oxidative stress-induced DNA damage was augmented after *sestrin2* siRNA treatment in the hippocampal CA1 subfield after TGI (Figure 3). Accordingly, the down-regulation of sestrin2 expression augments neuronal injury in the hippocampal CA1 subfield after TGI (Figure 4). Although we did not show direct evidence between RpS6 and oxidative stress in this study, it was known that the down-regulation of p-RpS6 by *RpS6* siRNA abrogate the protective ability against oxidative stress in ischemic heart conditions [13]. Thus, at least partly, sestrin2 may exert an antioxidant effect through p-RpS6 under the cerebral ischemic condition.

## 3. Experimental Section

### 3.1. Animals and Transient Global Ischemia

The experimental procedures conformed to the guidelines of our institutional committee on experimental animals in this study. Male adult Sprague-Dawley rats (250–325 g) were purchased from BioLASCO Taiwan Co., Ltd. (Taipei, Taiwan). They were housed in an Association for Assessment and Accreditation of Laboratory Animal Care (AAALAC) International-accredited animal facility under temperature control (24–25 °C) and 12 h light-dark cycle. Standard laboratory rat chow and tap water were available *ad libitum*. Rats were first anesthetized with chloral hydrate (400 mg/kg, i.p.) to perform preparative surgery. The TGI model was performed as previously reported [20,21].

### 3.2. siRNA Administration

Microinjection of siRNA into the hippocampal CA1 subfield was performed bilaterally using a stereotaxically positioned 27-gauge stainless steel needle connected to a 0.5 μL Hamilton microsyringe (Hamilton Company, Reno, NV, USA) as previously reported [17,19,21]. To inhibit sestrin2 expression, we used pre-designed sestrin2 siRNA from MISSION^®^ siRNA (Sigma-Aldrich Ltd., St. Louis, MO, USA); the sequences were as follows: sense, 5ʹ-CAGAGUAUUGUAACAU-3ʹ, antisense, 5ʹ-AUAGUGUUACAAUACUCUG-3ʹ. For negative control siRNA(NC), the sequences were as follows: 5ʹ-GAUCAUACGUGCGAUCAGA-3ʹ, antisense, 5ʹ-UCUGAUCGCACGUAUGAUC-3ʹ. The final concentration of siRNA was 0.05 nM in a total volume of 400 nL for injection into each side of the hippocampal CA1 subfield 24 h before TGI.

### 3.3. Tissue Sample Collection from the Hippocampus

At pre-designed time intervals (1, 4, 24, or 48 h) after TGI, rats were again anesthetized and perfused transcardially with 50 mL of warm (37.8 °C) saline containing heparin (100 U/mL) as previously reported [39,40,41]. The brain was taken out quickly under visual check and then placed on a piece of gauze moistened with ice-cold 0.9% saline. Tissues from the bilateral hippocampal CA1 area were collected and these samples were stored at −80 °C until use [17,19,21]. The protein concentration from the hippocampal CA1 tissue samples were measured with the BCA Protein Assay (Pierce, Rockford, IL, USA).

### 3.4. Western Blotting

Samples (20–40 μg of protein) were resolved through 2% SDS/polyacrylamide gels and transferred to PDVF membranes as previously reported [17,19,21]. The membranes were incubated with a primary anti-sestrin2 antibody (Abcam, Cambridge, MA, USA), phospho-RpS6 (Ser235/236), RpS6 antibody (Cell Signaling, Danvers, MA, USA), or mouse monoclonal antiserum against á-tubulin (Santa Cruz Biotechnology, Santa Cruz, CA, USA) for 1–2 h at room temperature. The specific antibody-antigen complex was detected and measured semiquantitatively as previously reported [17,19,21]. The color reaction was developed by the Blot AP System from Promega (Fitchburg, WI, USA).

### 3.5. Immunohistochemical Staining

After TGI and reperfusion, rats were anesthetized with pentobarbital (100 mg/kg, i.p.) and perfused transcardially with 150 mL of physiological saline; then followed by perfusion 400 mL of 4% paraformaldehyde in 0.1 M PBS for tissue fixation. The forebrains containing the dorsal hippocampus were dissected carefully. The brain sections were then blocked and fixed with additional 2 h in the same perfusion fixatives and thereafter transferred to a solution containing 30% sucrose in 0.1 M PBS for immunohistochemical staining. The brain sections embedded in tissue freezing medium (Sakura Finetek, Torrance, CA, USA) were serially sectioned throughout the rostral-caudal extent of the hippocampus at 10 µm thickness on a cryostat, and mounted on Superfrost/plus slides. The sections were permeabilized with 0.3% Triton X-100/10% horse serum in 0.01 M PBS for 20 min then incubated with primary antibodies. A primary anti-8OH-dG antibody (Cell Signaling, Danvers, MA, USA) was then applied to the sections overnight at 4 °C. The next day, the brain sections were incubated with a secondary biotinylated antibody (Vector Laboratories, Burlingame, CA, USA) for 1 h. The sections were washed and then incubated with ABC Elite complex (Vector Laboratories) for 1 h. The immunostainings of anti-8OH-dG antibody were visualized with DAB (Sigma Co., St. Louis, MO, USA). Images were captured under an Olympus AX70 (Olympus, Tokyo, Japan) light microscope. The numbers of 8-OHdG-positive cells were counted in the length of 250 µm of the pyramidal cell layer, in the middle of the hippocampal CA1 subfield, which were denoted as 8-OHdG-positive cells/section.

### 3.6. Qualitative and Quantitative Analysis of DNA Fragmentation

To qualitatively evaluate DNA fragmentation, total DNA were extracted from the hippocampal tissues and nucleosomal DNA ladders were amplified by a PCR kit (Maxim Biotech, San Francisco, CA, USA); then the samples were separated by 1% agarose gel electrophoresis. To quantitatively evaluate DNA fragmentation, a cell death enzyme-linked immunosorbent assay (Roche Molecular Biochemicals, Mannheim, Germany) was used to assess the histone-associated DNA fragments in the cytoplasm as previously reported [17,19].

### 3.7. Terminal Deoxynucleotidyl Transferase-Mediated dUTP-Biotin Nick End Labeling (TUNEL) Staining

The rats were processed for TUNEL staining 48 h after the onset of reperfusion following a 10 min episode of TGI with a commercial *in situ* apoptosis detection kit (ApopTag, Intergen Company, Purchase, NY, USA) as previously reported [17]. The TUNEL-positive cells were counted with the length of 1 mm of the pyramidal cell layer, in the middle of the hippocampal CA1 subfield, using an Olympus AX70 microscope, and were stated as the TUNEL-positive cells/section.

### 3.8. Statistical Analysis

Results are expressed as mean ± SEM. One way analysis of variance followed by the Scheffe multiple range tests for *post-hoc* assessment of individual means were used to compare the group mean differences. A *p*-value less than 0.05 was considered significant.

## 4. Conclusions

In conclusion, this study showed that sestrin2 may function as an endogenous protective mechanism in cerebral ischemia by affecting p-RpS6 expression, modulating oxidative status, and influencing neuronal damage. Any measurement to enhance sestrin2 expression in cerebral ischemia should have clinical potential in counteracting ROS-related neuronal damage.
